# Prognostic impact of biomarkers in PMBCL: rationale for early integration of immune checkpoint inhibitors

**DOI:** 10.37349/etat.2025.1002318

**Published:** 2025-05-20

**Authors:** Yana K. Mangasarova, Runiza R. Abdurashidova, Natalya V. Risinskaya, Bella V. Biderman, Tatiana V. Abramova, Vadim L. Surin, Irina A. Shupletsova, Tatiana N. Obukhova, Rasul I. Iusupov, Yulia A. Chabaeva, Aminat U. Magomedova, Lena E. Nikulina, Sergei M. Kulikov, Eugene E. Zvonkov, Alla M. Kovrigina, Andrey B. Sudarikov

**Affiliations:** University of Salford, UK; ^1^National Medical Research Center for Hematology, 125167 Moscow, Russian Federation; ^2^Lomonosov Moscow State University, 119991 Moscow, Russian Federation; ^3^I.M. Sechenov First Moscow State Medical University (Sechenov University), 119991 Moscow, Russian Federation

**Keywords:** PMBCL, immune checkpoint inhibitors, loss of heterozygosity, genomic instability

## Abstract

**Aim:**

This research aims to guide future strategies for personalized treatment of primary mediastinal large B-cell lymphoma (PMBCL), particularly to identify high-risk patients who may benefit from incorporating immune checkpoint inhibitors (ICIs) in the first-line setting.

**Methods:**

A retrospective, single-center study included 254 newly diagnosed PMBCL patients treated with rituximab, dose-adjusted etoposide, prednisone, vincristine, cyclophosphamide, doxorubicin (R-DA-EPOCH), rituximab, modified protocol NHL-BFM-90 (RmNHL-BFM-90), or R-DA-EPOCH combined with nivolumab. Clinical parameters, immunohistochemical markers [programmed death ligand-1 (PD-L1), programmed death-1 (PD-1), cytotoxic T-lymphocyte-associated protein 4 (CTLA-4), human leucocyte antigen (HLA)-DR, Ki-67, multiple myeloma oncogene 1 (MUM1)], molecular markers (mutations in tumor protein p53 (*TP53*), *CD58*, beta-2-microglobulin (*B2M*), and exportin 1 (*XPO1*) genes; short tandem repeats at 6p21.3 [major histocompatibility complex (*MHC*) class I/II], 9p24.1 (*PD-L1*/*PD-L2*), 16p13.13 [class II, MHC, transactivator gene (*CIITA*)]), and cytogenetic profiles [myelocytomatosis oncogene (*MYC*)/8q24, B-cell lymphoma 2 (*BCL2*)/18q21, *BCL6*/3q27, del17p13, and karyotype abnormalities] were analyzed.

**Results:**

The addition of nivolumab to R-DA-EPOCH as a first-line regimen significantly improved event-free survival (EFS; *P* = 0.018). This study identified that adverse prognostic factors for PMBCL include allelic imbalance at specific loci 6p21.3 (*MHC* class I/II), 9p24.1 (*PD-L1*/*PD-L2*), and 16p13.13 (*CIITA*). Incorporating nivolumab into the R-DA-EPOCH regimen as a first-line therapy has shown potential in reducing adverse prognostic factors.

**Conclusions:**

These findings suggest that high-risk patients may benefit significantly from the early incorporation of ICIs into their treatment plans.

## Introduction

Primary mediastinal large B-cell lymphoma (PMBCL) is a rare and aggressive subtype of diffuse large B-cell lymphomas (DLBCL), comprising 2–3% of all non-Hodgkin lymphomas and 7–10% of DLBCL cases [1, 2]. It primarily affects young adults and shows a slight female predominance [3]. Due to the young age of affected patients, minimizing long-term treatment-related toxicity, especially concerning fertility and quality of life, is a clinical priority.

Standard treatment regimens, particularly rituximab, dose-adjusted etoposide, prednisone, vincristine, cyclophosphamide, doxorubicin (R-DA-EPOCH), have resulted in high complete remission (CR) rates and long-term survival in over 90% of patients [4, 5]. However, relapsed or refractory (R/R) cases remain challenging, with limited options and poor outcomes [6–8]. Existing clinical predictors, such as lactate dehydrogenase (LDH) level, Eastern Cooperative Oncology Group (ECOG) performance status, and extranodal involvement, have shown inconsistent prognostic value [9–11]. Furthermore, well-established molecular markers like tumor protein p53 (*TP53*) or B-cell lymphoma 2 (*BCL2*) gene mutations have not demonstrated reliable prognostic significance in PMBCL [12, 13], underscoring the need for new biomarkers to guide risk stratification and therapy.

Immune checkpoint inhibitors (ICIs) have emerged as a promising treatment option for R/R PMBCL. The KEYNOTE-170 trial demonstrated that pembrolizumab treatment provided an overall response rate of 41.5% (20.8% CR) in heavily pretreated patients, with durable remissions observed [14]. Despite these encouraging results, the use of ICIs in the first-line setting or in combination with chemotherapy for PMBCL remains underexplored [15].

The unique immunobiological features of PMBCL underpin its pathogenesis and responsiveness to ICIs. A hallmark of PMBCL is the amplification of the 9p24.1 locus, which leads to overexpression of the immune checkpoint ligands programmed death ligand-1 (PD-L1) and PD-L2 and facilitates immune evasion across T-cell anergy [16, 17]. In addition to immune checkpoint activation, PMBCL frequently exhibits reduced tumor immunogenicity due to impaired antigen presentation. This is associated with structural alterations and mutations in key regulators of major histocompatibility complex (MHC) expression, including class II, MHC, transactivator gene (*CIITA*) and human leucocyte antigen (*HLA*) genes.

Structural genomic abnormalities, including balanced chromosomal rearrangements, copy number gains and losses, and copy-neutral loss of heterozygosity (cnLOH), represent common markers in PMBCL [18, 19].

We hypothesize that microsatellite repeat aberrations flanking target genes may serve as indicators of broader genomic alterations and reflect chromosomal events affecting these genes. Evidence from colorectal and pancreatic cancers has demonstrated that chromosomal instability is reflected in microsatellite instability [20–22]. Therefore, in addition to clinical, immunohistochemical (IHC) [PD-L1, programmed death-1 (PD-1), cytotoxic T-lymphocyte-associated protein 4 (CTLA-4), HLA-DR, Ki-67, multiple myeloma oncogene 1 (MUM1)], and mutational analyses [*TP53*, *CD58*, beta-2-microglobulin (*B2M*), exportin 1 (*XPO1*)] as well as cytogenetic profiling [rearrangements in myelocytomatosis oncogene (*MYC*)/8q24, *BCL2*/18q21, *BCL6*/3q27, del17p13, and karyotype abnormalities], we propose to analyze microsatellite markers. Therefore, we have developed the panel to assess short tandem repeat (STR) profiles in key genomic regions, including 6p21.3 (*MHC* class I/II), 9p24.1 (*PD-L1*/*PD-L2*), and 16p13.13 (*CIITA*). In this study, we aimed to explore the prognostic and predictive significance of clinical, molecular, and IHC markers in a large cohort of PMBCL patients treated with R-DA-EPOCH or rituximab, modified protocol NHL-BFM-90 (RmNHL-BFM-90) protocols at a single center to identify high-risk patients who may benefit from incorporating ICIs in the first-line setting.

## Materials and methods

Patients with newly diagnosed PMBCL confirmed by WHO criteria (*n* = 254) who attended National Medical Research Center for Hematology (Moscow, Russian Federation) from November 2007 to July 2024 were included in the retrospective single-center study. Eligibility criteria included no prior systemic therapy before enrollment. From 2007 to 2013, patients received treatment according to the RmNHL-BFM-90 protocol, whereas from 2013 to 2022, the R-DA-EPOCH protocol was adopted [11, 23]. In 2023, a randomized protocol comparing nivolumab in combination with R-DA-EPOCH versus R-DA-EPOCH alone was initiated, and the results are yet to be determined (ClinicalTrials.gov identifier: NCT06188676). Patients received six cycles of induction therapy. Upon achieving a CR after these cycles, the treatment was concluded. Patients with partial remission (PR) underwent two additional courses of rituximab, cisplatin, dexamethasone, cytarabine (R-DHAP), followed by autologous hematopoietic stem cell transplantation (auto-HSCT) using lomustine, etoposide, cytarabine, melphalan (CEAM) [11].

**Figure 1:**
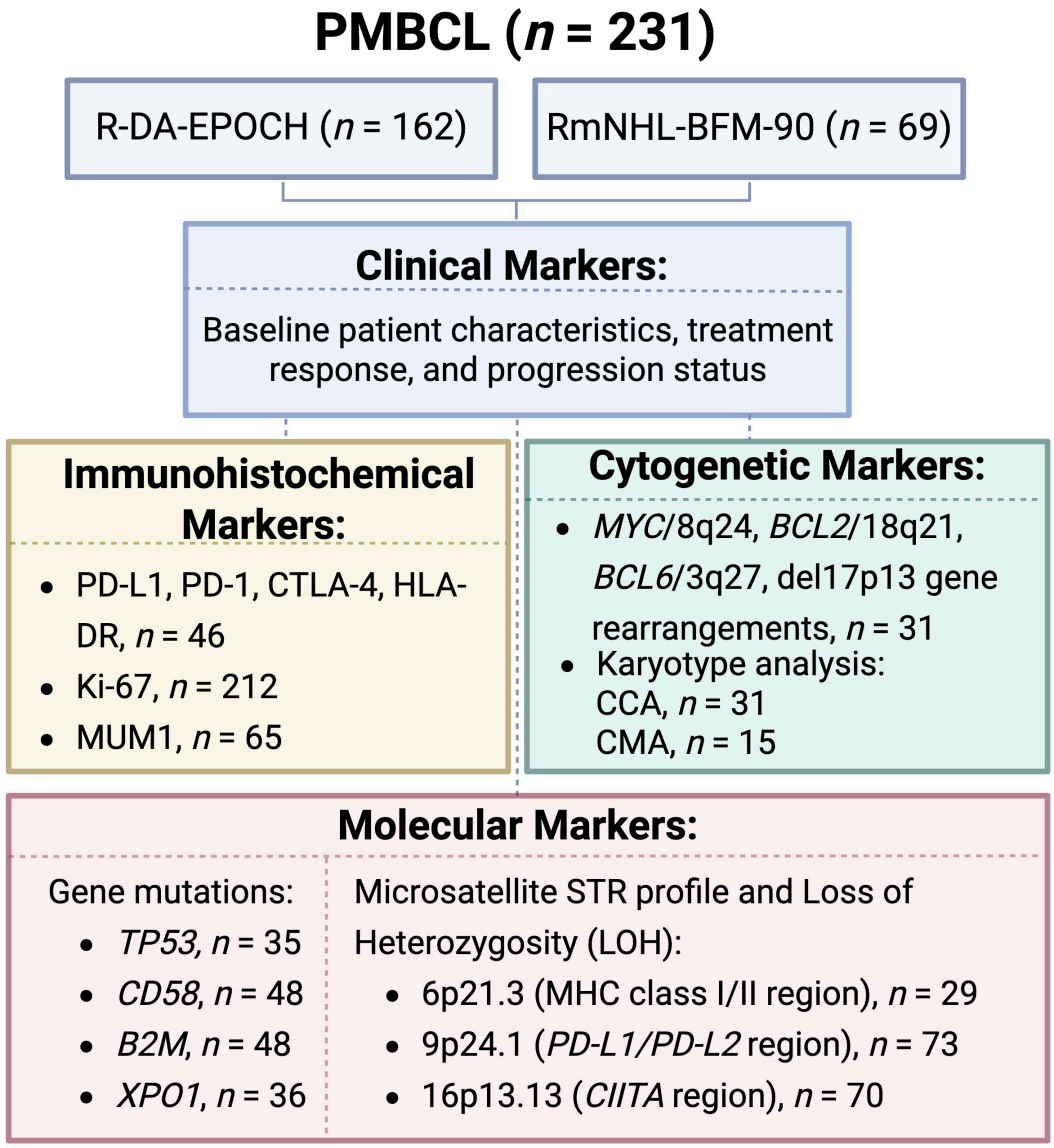
**Schematic overview of the study design**. PMBCL: primary mediastinal large B-cell lymphoma; R-DA-EPOCH: rituximab, dose-adjusted etoposide, prednisone, vincristine, cyclophosphamide, doxorubicin; RmNHL-BFM-90: rituximab, modified protocol NHL-BFM-90; PD-L1: programmed death ligand-1; PD-1: programmed death-1; CTLA-4: cytotoxic T-lymphocyte-associated protein 4; HLA: human leucocyte antigen; MUM1: multiple myeloma oncogene 1; *MYC*: myelocytomatosis oncogene; *BCL2*: B-cell lymphoma 2; CCA: conventional cytogenetic analysis; CMA: chromosome microarray analysis; *TP53*: tumor protein p53; *B2M*: beta-2-microglobulin; *XPO1*: exportin 1; STR: short tandem repeat; MHC: major histocompatibility complex; *CIITA*: class II, MHC, transactivator gene

A comprehensive analysis of the R-DA-EPOCH and RmNHL-BFM-90 cohorts (*n* = 231) was conducted to assess clinical parameters, IHC markers (PD-L1, PD-1, CTLA-4, HLA-DR, Ki-67, MUM1), and molecular alterations (mutations in *TP53*, *CD58*, *B2M*, and *XPO1* genes). STR profiles in key genomic regions including 6p21.3 (*MHC* class I/II), 9p24.1 (*PD-L1*/*PD-L2*), 16p13.13 (*CIITA*), and cytogenetic profiles (*MYC*/8q24, *BCL2*/18q21, *BCL6*/3q27, del17p13 rearrangements, and karyotype abnormalities) were also analyzed. Details of the study design are provided in Figure 1. This study has been reviewed and approved by the appropriate institutional review board and all patients involved have provided informed consent.

### Immunohistochemistry

Samples were analyzed using antibodies to PD-L1 (28–2, CELL MARQUE, USA), HLA-DR (TAL.1B5, Dako, Denmark), PD-1 (NAT.105, CELL MARQUE, USA), CTLA-4 (CAL49, Abcam, UK), MUM1 (EAU32, Leica), and Ki-67 (K2, Leica). IHC procedures were performed on tumor biopsies: mediastinal masses (*n* = 190, 90%), lymph nodes (*n* = 12, 6%), and extranodal sites of involvement (*n* = 10, 5%). For each sample, 4 μm slices were stained using a standard immunohistochemistry protocol for formalin-fixed paraffin-embedded (FFPE) tissues, with a ready-to-use detection system that provides high signal amplification without biotin, utilizing the Leica Bond-MAX immunostainer. Surgipath Sub-X Leica medium was used as the final mounting medium. Antibody dilutions and the type of buffer for epitope retrieval (ER)—ER1 (pH = 6) or ER2 (pH = 9)—were optimized experimentally in advance. The expression of PD-L1, HLA-DR, MUM1, and Ki-67 was assessed in CD20+ tumor cells. The reaction was considered reliable in the presence of a positive control—small T-cells and macrophages. The threshold value was set at 50% positive large tumor cells, while PD-1 and CTLA-4 expression were evaluated in CD3+ T-cells within the tumor microenvironment.

### Fluorescence *in situ* hybridization

Fluorescence *in situ* hybridization (FISH) was performed on 31 tumor biopsy samples to detect chromosomal aberrations involving *MYC*/8q24 (*n* = 31), *BCL2*/18q21 (*n* = 31), *BCL6*/3q27 (*n* = 31), and del17p13 (*n* = 16) loci. Standard protocols were used, and preparations were analyzed using an Axio Imager Z2 (Carl Zeiss, Germany) fluorescence microscope with result documentation performed using the ISIS imaging system (MetaSystems, Germany). At least 200 interphase nuclei with high-quality signals were assessed for each sample.

### Conventional cytogenetic analysis

Conventional cytogenetic analysis (CCA) involved short-term culture of homogenized cell suspensions from 31 biopsy material samples. Karyotypes were analyzed using a Zeiss Axioscope microscope equipped with the IKAROS imaging system. A minimum of 20 metaphase spreads were analyzed per sample to detect chromosomal abnormalities.

### Chromosome microarray analysis

The CytoScan™ HT-chromosome microarray analysis (CMA) 96F array SNP-oligonucleotide microarray was used for the analysis, which was performed by the Genomed Laboratory of Molecular Pathology in Moscow, Russia. The samples were DNA isolated from 15 mediastinal tumor biopsy samples, with a quantity ranging from 100 ng to 200 ng, and an A260/A280 ratio of at least 1.8. The results were processed using the Multi Sample Viewer Software (v.1.1.0.11) and Chromosome Analysis Suite (ChAS 4.3.0.71) (Thermo Fisher Scientific, USA). The cutoff for a CNA size was set at ≥ 5 Mb, following the guidelines of Schoumans et al. [24].

### *CD58* and *B2M* mutation analysis

Mutations in the *CD58* and *B2M* genes were evaluated in a subset of 48 patients. DNA was extracted from tumor biopsies by standard proteinase K-SDS digestion and phenol-chloroform extraction [25]. We analyzed functionally important regions of the *CD58* and *B2M* genes, i.e. the promoter region, exons 1–2 of the *B2M* gene, and exons 1–3 of the *CD58* gene and exon-intron junctions, by Sanger sequencing. For the amplification of target fragments, we used primers designed in the laboratory of genetic engineering at our center (Table 1).

**Table 1:** Primer sequences for *B2M* and *CD58* PCR

**Name**	**Primer sequences (5'–3')**	**Location**	**PCR product size (bp)**
Gene *B2M*			
*B2M1D*	CAGACAGCAAACTCACCCAGT	Exon 1	451
*B2M1R*	CTTCCCCGAGATCCAGCCCT		
*B2M2Dx*	CTTGACACCAAGTTAGCCCCA	Exon 2	510
*B2M2Rx*	GAACATTCCCTGACAATCCCA		
Gene *CD58*			
*CD58D1*	GGAGCCCTACTTCTGGCCGA	Exon 1	276
*CD58R1*	CCGTCCCCACCCGTCTCTGA		
*CD58D2x*	GTGTCAGCAGTTTGTCAGCT	Exon 2	517
*CD58R2x*	CCCTGACAACAGGTAACATCT		
*CD58D3*	GGAGTTTGTCTGCTCATCCT	Exon 3	450
*CD58R3*	GAACCTTGTGTTAGTCACCACA		

D: forward primers; R: reverse primers. *B2M*: beta-2-microglobulin; PCR: polymerase chain reaction

Polymerase chain reaction (PCR) was carried out using PCR Master Mix (Thermo Fisher Scientific, USA) following the manufacturer’s protocol. PCR products were analyzed by electrophoresis in 1.5% agarose gel and then purified using Wizard PCR Preps DNA purification system (Promega, USA). To determine the nucleotide sequence of genes, Sanger sequencing was performed using the ABI PRISM^®^ BigDyeTM Terminator v.3.1 kit (Thermo Fisher Scientific, USA) on a Nanofor-05 automatic genetic analyzer (Syntol, Russian Federation) according to the manufacturer’s protocols. The obtained nucleotide sequences were compared with the corresponding reference sequences from the NCBI database (B2M: NM_004048.4; NG_012920.2; CD58: NM_001779.3) [26]. The results of Sanger sequencing were described in accordance with the recommendations of the Human Genome Variation Society (HGVS) [27]. The pathogenicity of variants was assessed using the following prediction tools [SIFT v.6.2.1 (J. Craig Venter Institute) [28], PROVEAN v.1.1.5 (J. Craig Venter Institute) [29], PolyPhen-2 v.2.2.2 (Harvard Medical School) [30], and MutationTaster (Charité – Universitätsmedizin Berlin) [31]] and ClinVar (NCBI, U.S. National Library of Medicine) [32].

### *TP53* mutation analysis

Mutations in the *TP53* gene were analyzed in a subset of 35 patients. DNA was extracted from tumor biopsies or sections of FFPE tissue blocks [33]. Exons 4 through 10 of the *TP53* gene were amplified in five separate PCR reactions. Library preparation was performed using the Nextera XT DNA (Illumina, San Diego, CA, USA), followed by sequencing on a MiSeq genetic analyzer (Illumina, USA). Bioinformatics analysis was conducted using a pipeline that included tools such as Trimmomatic [34], BWA [35], SAMtools [36], VarDict [37], and Annovar [38]. The identified variants were further assessed for potential pathogenicity using the Franklin by Genoox platform [39] and the SESHAT [40] online databases.

### *XPO1* mutation analysis

The analysis of the E571 mutations (predominantly E571K or E571G) in the *XPO1* gene was performed on tumor biopsy DNA samples from 36 patients using allele-specific PCR (AS-PCR) on the CFX96 Touch Real-Time PCR Detection System from Bio-Rad Laboratories, Inc. (USA). The primers and probes used in this study are listed in Table 2.

**Table 2:** Primer sequences for *XPO1* qPCR

**Primer**	**Forward**	**Probe**	**Reverse**
*XPO1* wild type	GCATCAAATATCATGTACATAG	FAM-CAGAAATT(RTQ1)TCCAGTGAGCTCTCA-P	GAGATTTACCATGCATGAATTC
*XPO1*-E571K/E571G	GCATCAAATATCATGTACATAG	FAM-CAGAAATT(RTQ1)TCCAGTGAGCTCTCA-P	GAGATTTACCATGCATGAATTK^※^

^※^ K (G or T) (https://www.bioinformatics.org/sms/iupac.html). *XPO1*: exportin 1; qPCR: quantitative polymerase chain reaction

The PCR conditions for TaqMan real-time AS-PCR included preheating at 94°C for 300 s, followed by 45 cycles of thermal cycling. The denaturation step was at –94°C for 20 s, while the annealing and elongation steps were at –60°C for 50 s. Each primer was used at a concentration of 10 pmole per reaction, and each probe was used at 5 pmole per reaction. The reaction volume was 25 mL and the PCR buffer, magnesium chloride, dNTPs, and Taq polymerase were provided by Syntol LLC (Moscow, Russia). Primers and TaqMan probes were synthesized at Syntol according to the authors’ design.

### STR-profiling

STR profiles of the tumor cell DNA were analyzed on a cohort of 93 patients. A diagnostic system for the investigation of allelic imbalance (AI) in microsatellite repeats located near the *PD-L1*/*PD-L2* genes (loci 9p24.1) and *CIITA* genes (loci 16p13.13) using the STR-PCR method has been developed. The methodology has been described in detail previously [41]. The primers for microsatellite repeats located near the *HLA* (loci 6p21.3) were adopted from the publication by Chambuso et al. [42] (2019).

Tumor DNA was isolated from the biopsy samples taken at diagnosis. Control DNA was isolated from the blood samples collected during CR or from bone marrow without tumor involvement. AI was assessed by comparing heterozygous markers from tumor DNA with those of matched control DNA. Patients exhibiting homozygous inheritance for any of the studied markers were excluded from further analysis due to the inability to evaluate AI.

AI of microsatellite repeats was examined in the regions of *HLA* [loci 6p21.3, (GT)n and (CA)m], *PD-L1*/*PD-L2* [loci 9p24.1, (GT)n and (TTAT)m] and *CIITA* [loci 16p13.13, (CA)n and (GT)m] using STR-PCR (Table 3) with fragment analysis. Six separate PCR reactions with specific primers to amplify target loci markers on a DNAEngine thermal cycler (Bio-Rad, USA) were used. PCR products were then subjected to capillary electrophoresis using the Nanophor-05 genetic analyzer (Syntol LLC, Russia), and the data were analyzed using GeneMarker software, version 3.0.1 (SoftGenetics, USA).

**Table 3:** Primer sequences for STR-PCR

**Primer**	**Forward**	**Reverse**
6p21.3 (GT)n	GCAACTTTTCTGT	FAM-ACCAAACTT
	CAATCCA	CAAATTTTCGG
6p21.3 (CA)m	ACGTTCGTACCC	FAM-ATCGAGGTA
	ATTAACCT	AACAGCAGAAA
9p24.1 (GT)n	TCCATGTTGCCA	FAM-GAGGCTGTG
	CAAATGACA	GGTGGGACGAT
9p24.1 (TTAT)m	GGCATCTGCTTT	FAM-AGTAGTGAG
	GACCATGA	CCGAGATCTTG
16p13.13 (CA)n	FAM-TGCATTGT	СATAACCACGCAC
	TGCATCCAGCCT	GCACCCT
16p13.13 (GT)m	FAM-CCAGCCCA	CCTGGTCAAAAAA
	GCACTGTGACCT	CATGCCA

STR: short tandem repeat; PCR: polymerase chain reaction

### Statistical analyses

Categorical variables were analyzed using Fisher’s exact test for small sample sizes and the *χ*^2^ test when the minimum expected value for all categories exceeded 5. Continuous variables were assessed using nonparametric methods, including the Mann-Whitney *U* test for comparisons between two groups and the Kruskal-Wallis *H* test for comparisons among three groups. Survival analysis was conducted using the Kaplan-Meier method to estimate survival, with group comparisons performed using the log-rank test. Odds ratios (OR) with corresponding 95% confidence intervals (CI) were calculated to evaluate the association between binary categorical variables and outcomes. OR were computed using contingency tables. Fisher’s exact test or *χ*^2^ test was used to assess the statistical significance of these associations, depending on sample size and cell frequencies. Overall survival (OS) was defined as the time from the date of diagnosis to death from any cause. Event-free survival (EFS) was calculated as the time from the initiation of chemotherapy to the earliest occurrence of relapse, disease progression, and switch to alternative anti-cancer therapy due to refractory disease or PR. All statistical analyses were performed using R version 4.1 (R Core Team, 2017). Statistical significance was defined as *P* < 0.05.

## Results

The demographic and clinical characteristics of the patient sample are presented in Table 4.

**Table 4:** Baseline clinical characteristics of PMBCL patients by treatment group

**Characteristic**	**R-DA-EPOCH (*n* = 162)**	**RmNHL-BFM-90 (*n* = 69)**	**R-DA-EPOCH + nivolumab (*n* = 23)**	***P* ^※^**
Median age (range), years	32 (19–69)	29 (19–68)	31 (20–58)	0.111
≥ 45 years, *n* (%)	25 (15)	9 (13)	4 (17)	0.956
Male/female, *n* (%)	50/112 (31/69)	25/44 (36/64)	9/14 (39/61)	0.592
Ann Arbor stage				
I–II, *n* (%)	132 (81)	64 (93)	17 (74)	0.041
III–IV, *n* (%)	30 (19)	5 (7)	6 (26)	
ECOG-PS, *n* (%)				
≥ 2	141 (87)	63 (91)	20 (87)	0.643
Bulky mass ≥ 6 cm, *n* (%)	151 (93)	67 (97)	22 (96)	0.479
Bulky mass ≥ 12 cm, *n* (%)	67 (41)	21 (30)	13 (57)	0.063
Involvement of pleura/pericardium, *n* (%)	116 (72)	48 (70)	18 (78)	0.725
Involvement of soft tissues/breast tissue, *n* (%)	38 (23)	15 (22)	6 (26)	0.907
Bone marrow involvement, *n* (%)	3 (2)	1 (1)	0	0.234
Elevated lactate dehydrogenase (N < 247 UI/L), *n* (%)	144 (89)	60 (87)	20 (87)	0.900
IPI score				
0–1, *n* (%)	28 (17)	17 (25)	7 (30)	0.261
2, *n* (%)	98 (60)	41 (59)	9 (39)	
3, *n* (%)	34 (21)	9 (13)	6 (26)	
4–5, *n* (%)	2 (1)	2 (3)	1 (4)	
Extramediastinal involvement, *n* (%)	30 (19)	5 (7)	6 (26)	0.041

This table summarizes the baseline clinical and demographic characteristics of PMBCL patients stratified by treatment protocol. ^※^ The *P*-value of comparison between the treatment protocols. PMBCL: primary mediastinal large B-cell lymphoma; IPI: International Prognostic Index; R-DA-EPOCH: rituximab, dose-adjusted etoposide, prednisone, vincristine, cyclophosphamide, doxorubicin; RmNHL-BFM-90: rituximab, modified protocol NHL-BFM-90; ECOG: Eastern Cooperative Oncology Group; PS: performance status

**Figure 2:**
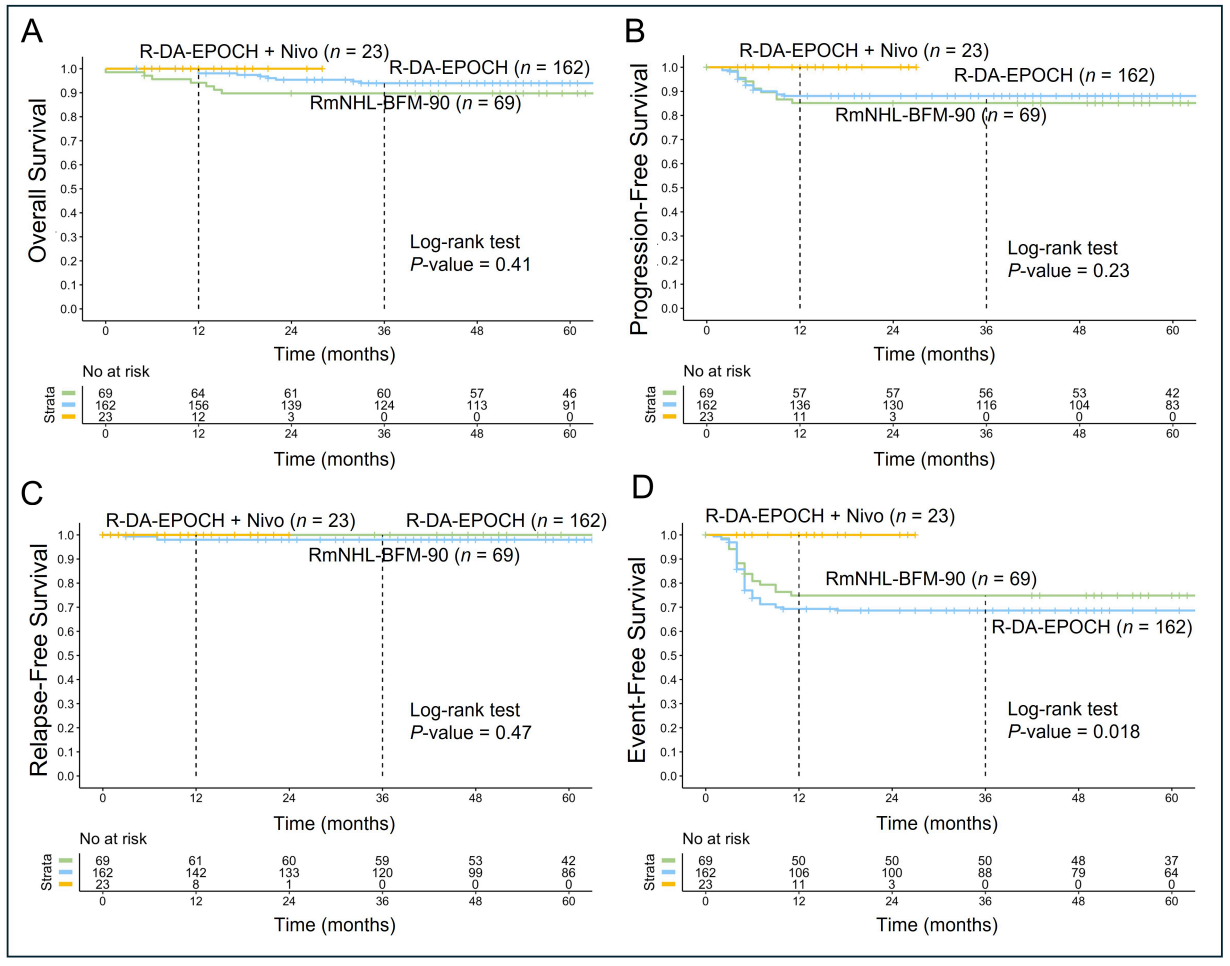
**Kaplan-Meier survival analysis of PMBCL patients treated with R-DA-EPOCH (*n* = 162), RmNHL-BFM-90 (*n* = 69), and R-DA-EPOCH with nivolumab (*n* = 23)**. (**A**) The analysis showed no statistically significant differences in OS between the groups (*P* = 0.41); (**B**) PFS analysis reveals a similar trend favoring R-DA-EPOCH with nivolumab, with no statistically significant difference (*P* = 0.23); (**C**) the RFS outcomes are comparable across the three treatment groups, with no significant differences observed (*P* = 0.47); (**D**) the EFS demonstrates a statistically significant improvement in the R-DA-EPOCH with nivolumab group (*P* = 0.018), suggesting that the addition of nivolumab reduces adverse events such as treatment failure, relapse, and progression requiring second-line therapy or auto-HSCT. PMBCL: primary mediastinal large B-cell lymphoma; R-DA-EPOCH: rituximab, dose-adjusted etoposide, prednisone, vincristine, cyclophosphamide, doxorubicin; RmNHL-BFM-90: rituximab, modified protocol NHL-BFM-90; OS: overall survival; PFS: progression-free survival; RFS: relapse-free survival; EFS: event-free survival; auto-HSCT: autologous hematopoietic stem cell transplantation

The addition of nivolumab to R-DA-EPOCH as a first-line therapy demonstrated a trend toward improved outcomes in OS, progression-free survival (PFS), and relapse-free survival (RFS). A statistically significant improvement was observed in EFS, reflecting fewer R/R and a reduced need for second-line therapy or hematopoietic stem cell transplantation (Figure 2). The median follow-up period was 71 months (range, 0–211 months). CR was achieved in 189 of 254 patients (74%), with early relapse involving central nervous system (CNS) observed in 3 of these patients (2%). PR was documented in 39 patients (15%), while disease progression occurred in 29 patients (11%). A total of 15 deaths (6%) were attributed to R/R disease. Additionally, one death during the first cycle of RmNHL-BFM-90 therapy was related to treatment toxicity, and another unrelated death occurred at 90 months due to a stroke.

Given the comparable survival outcomes between the R-DA-EPOCH and RmNHL-BFM-90 groups (OS: *P* = 0.32; PFS: *P* = 0.59; RFS: *P* = 0.27; EFS: *P* = 0.36), we have combined these groups to analyze clinical, molecular-cytogenetic, and IHC markers to identify predictors of poor prognosis that are unmitigated by standard therapies.

### Cytogenetic and molecular analyses, detected copy number abnormalities

**Figure 3:**
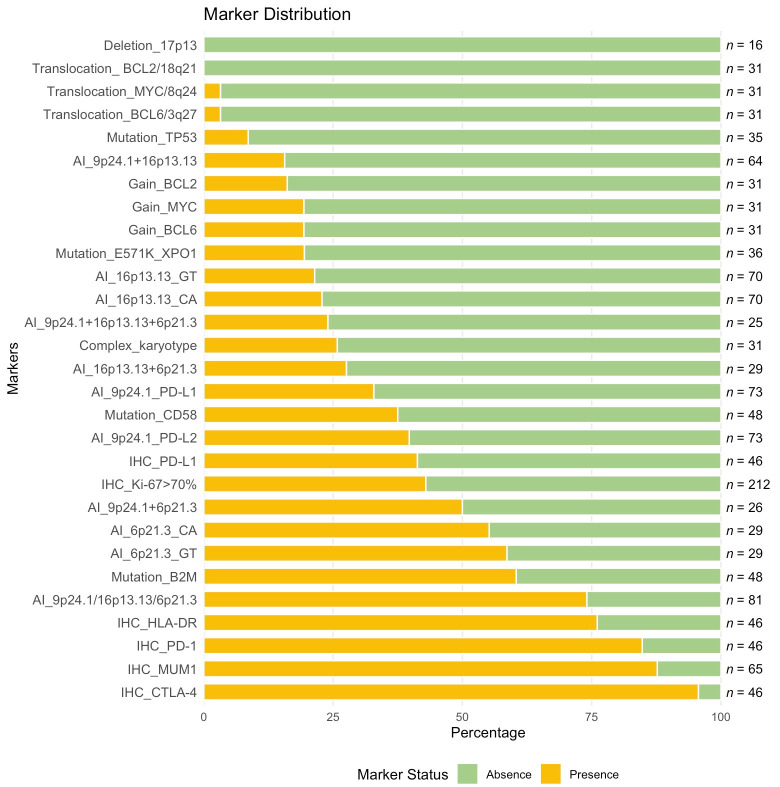
**Frequency of immunohistochemical, molecular, and cytogenetic markers in patients with PMBCL**. This figure illustrates the frequency distribution of negative (green, absence) and positive (yellow, presence) status of various markers in patients with PMBCL. Markers are sorted by their positive frequency, and the total number of patients analyzed for each marker is displayed on the right. PMBCL: primary mediastinal large B-cell lymphoma; BCL2: B-cell lymphoma 2; MYC: myelocytomatosis oncogene; TP53: tumor protein p53; XPO1: exportin 1; PD-L1: programmed death ligand-1; IHC: immunohistochemical; B2M: beta-2-microglobulin; HLA: human leucocyte antigen; PD-1: programmed death-1; MUM1: multiple myeloma oncogene 1; CTLA-4: cytotoxic T-lymphocyte-associated protein 4

CCA and FISH analyses of *MYC*, *BCL2*, and *BCL6* rearrangements were performed in 31 patients with PMBCL, while del17p13 was assessed in 16 patients. The frequency of detected markers is presented in Figure 3. Due to the low mitotic activity of tumor cells, standard cytogenetic analysis was challenging. Adequate mitoses were obtained in only 16 (52%) PMBCL samples, among which a complex karyotype was identified in 8 cases (50%). Translocations involving the *BCL6* and *MYC* loci were each observed once, whereas *BCL2* translocations and del17p13 were not detected. Copy gains (trisomy/duplication/amplification) of *MYC* and *BCL6* were identified in 6 cases (19%), while *BCL2* gains were observed in 5 cases (16%). The analyzed cytogenetic markers did not demonstrate any impact on the prognosis in PMBCL.

**Figure 4:**
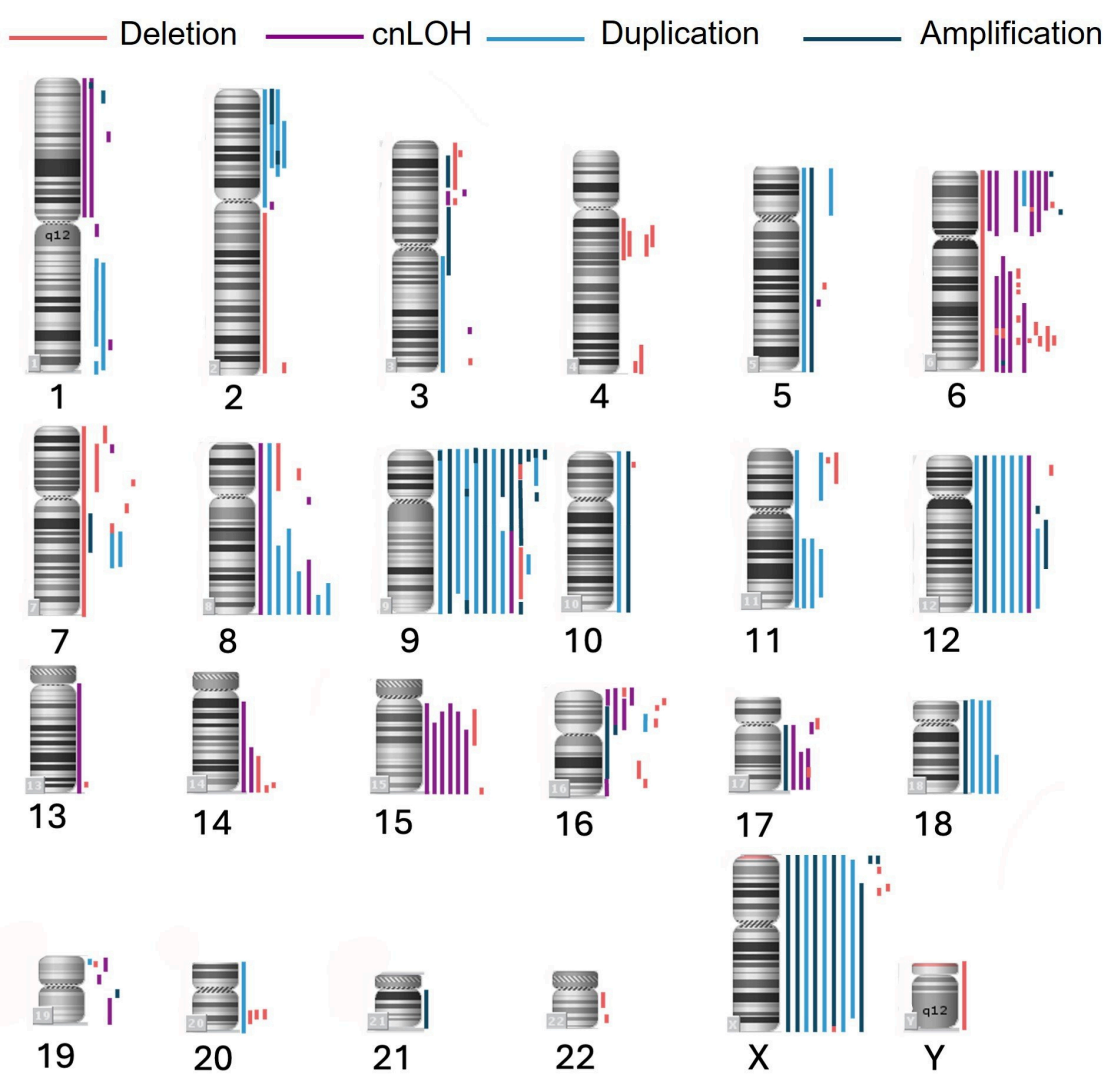
**Genome-wide distribution of copy number alterations and cnLOH in PMBCL samples (*n* = 15)**. This figure illustrates the chromosomal localization and frequency of genomic aberrations detected by CMA. Each vertical bar represents a distinct event across the cohort. Blue bars indicate copy number gains (amplifications or duplications), red bars denote deletions, and purple bars represent cnLOH. cnLOH: copy-neutral loss of heterozygosity; PMBCL: primary mediastinal large B-cell lymphoma; CMA: chromosome microarray analysis

CMA was performed in 15 patients with PMBCL. Genomic aberrations were detected in all analyzed cases, with a median number of aberrations of 15 (range, 6–25). The most frequent abnormalities were observed on chromosomes 6 and 9, detected in 13 out of 15 patients (87%). The predominant type of alteration was copy number gain, with a total of 96 such events recorded, of which 46% (44/96) were amplifications. Amplifications were most found on chromosome 9, occurring in 73% (11/15) of patients (15 events). In total, 68 deletions and 47 cases of cnLOH were identified. These aberrations most frequently affected chromosome 6: deletions were detected in 73% (11/15), and cnLOH in 60% (9/15) of cases. Biallelic loss was observed in locus 17q24.1. The localization and extent of the detected genomic aberrations are visualized in Figure 4. Focusing on loci of interest (9p24.1, 16p13.13, and 6p21.3), amplification of 9p24.1 was the most frequent aberration, identified in 13 (87%) cases. Quantitative abnormalities of the 6p21.3 locus were observed in 7 (47%) cases, including cnLOH in 5 (33%) cases, deletion in 1 (7%) case, and gain in 1 (7%) case. For the 16p13.13 locus, quantitative abnormalities were found in 6 (40%) cases, including cnLOH in 3 (20%) cases and deletion in 3 (20%) cases.

We have assessed microsatellite repeats flanking key genes (*PD-L1*/*PD-L2*, *CIITA*, and *HLA*) involved in immune evasion in PMBCL. The STR profile analysis included PMBCL patients with heterozygous inheritance for at least one marker of the pair. AI near the *PD-L1*/*PD-L2* (9p24.1) was detected in 24/73 (33%) and 29/73 (40%) patients, respectively. AI near the *CIITA* (16p13.13) was observed in 16/70 (23%) patients for the CA marker and in 15/70 (21%) for the GT marker. For the *HLA* (6p21.3), AI was identified in 16/29 (55%) patients for the CA marker and in 17/29 (59%) patients for the GT marker. The frequency of detected markers is presented in Figure 3.

The next step involved comparing the results obtained from STR-profile analysis with those from the CMA in 15 PMBCL patients. Aberrations larger than 5 Mb, along with microdeletions, were analyzed in accordance with the guidelines for genomic array analysis in acquired hematological neoplastic disorders [23]. In this exploratory study, we evaluated microdeletions, microduplications, and cnLOH sites to assess the inclusiveness of genes potentially involved in the pathogenesis of PMBCL. High-resolution CMA of tumor DNA revealed that amplification, pseudo-hyperdiploidy, and cnLOH manifest as AI. Thus, AI near the regions of interest may reflect underlying genomic instability. Allelic loss can result from either absolute loss of DNA content or copy-neutral loss of a parental allele. However, while STR analysis can identify the involvement of specific loci in pathogenesis, it cannot determine the precise chromosomal event (e.g., deletion or duplication) leading to AI.

### Molecular analysis: mutations in *TP53*, *CD58*, *B2M,* and *XPO1* genes

Mutations in *TP53* gene were detected in 4/35 (11%) patients. In 3 (9%) patients, the identified mutations were classified as pathogenic according to online databases. Notably, one tumor sample exhibited a rare combination of two pathogenic mutations in cis configuration, located in proximity, which may indicate a unique mechanistic feature. In the fourth patient, the mutation identified was classified as a variant of uncertain significance, and its potential impact on disease progression could not be determined. Consequently, this case was excluded from survival analyses. Mutations in the *B2M* gene were detected in 29 out of 48 patients (60%), representing the most frequently altered gene in this cohort. Mutations in the *CD58* gene were observed in 18 out of 48 patients (38%). E571K mutation in *XPO1* gene was identified in 7 out of 36 patients (19%). The frequency of detected markers is presented in Figure 3.

### Immunohistochemistry results

IHC analysis of key markers was performed to evaluate their expression in PMBCL tumor samples. The results are presented in Figure 3. Expression of PD-L1 was observed in 41% of samples (*n* = 46). The PD-1 receptor was detected in 85% of samples (*n* = 46). Expression of CTLA-4 was identified in 96% of tumor samples (*n* = 46). HLA-DR positivity was observed in 76% of samples. MUM1 was present in 88% of samples (*n* = 65). A high proliferation index (Ki-67 > 70%) was detected in 43% of samples (*n* = 212).

### Identification of poor outcome predictors

**Figure 5:**
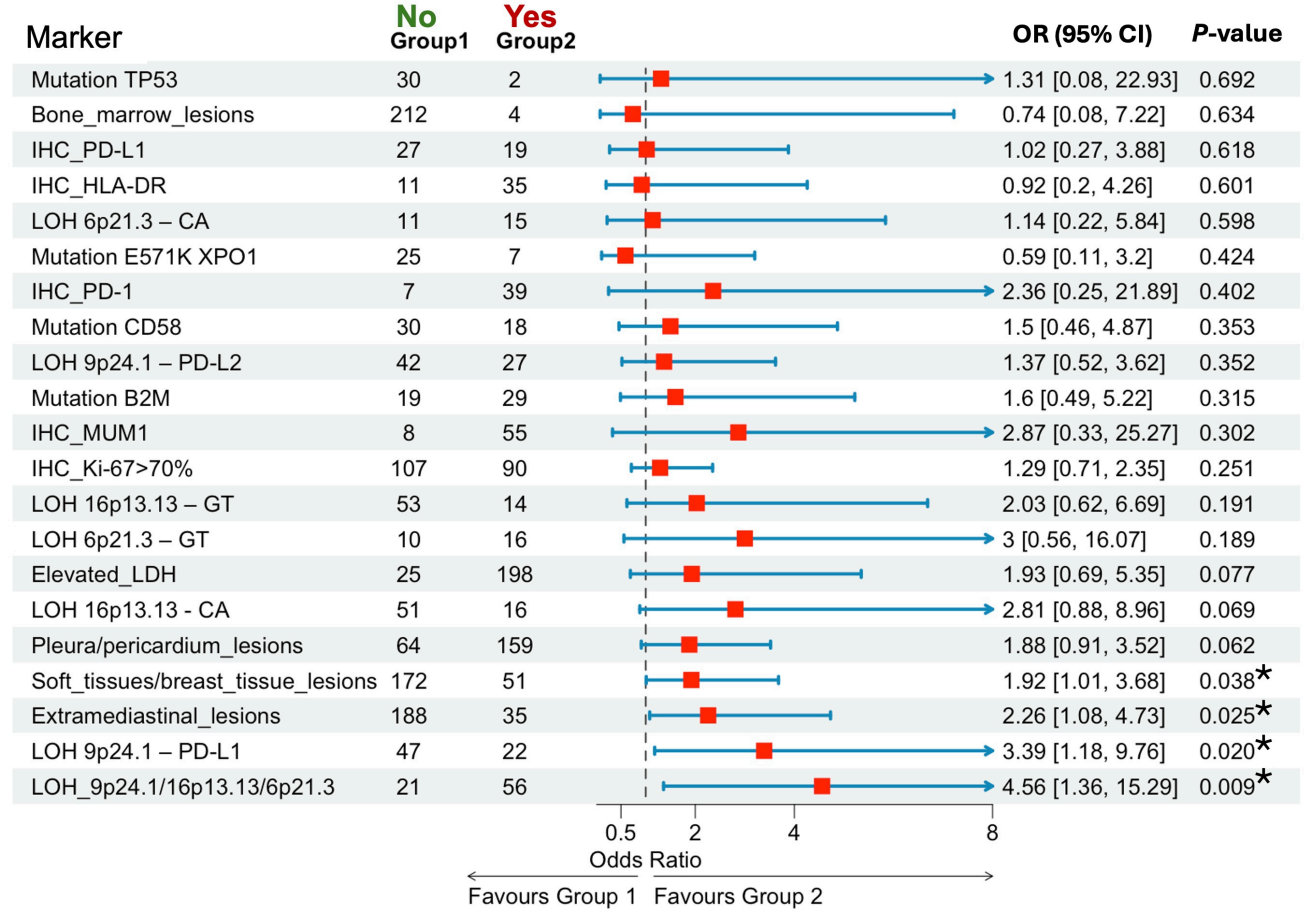
**Association of clinical, immunohistochemical, and molecular markers with poor outcomes (R/R or PR)**. ^*^ *P* < 0.05. R/R: relapsed or refractory; PR: partial remission; OR: odds ratios; CI: confidence intervals; TP53: tumor protein p53; IHC: immunohistochemical; HLA: human leucocyte antigen; LOH: loss of heterozygosity; XPO1: exportin 1; PD-1: programmed death-1; PD-L1: programmed death ligand-1; B2M: beta-2-microglobulin; MUM1: multiple myeloma oncogene 1; LDH: lactate dehydrogenase

A comprehensive analysis of clinical, IHC, and molecular markers was conducted. The initial step involved frequency analysis to evaluate potential predictors (Figure 5). Given the low number of fatal outcomes, OS analysis did not yield conclusive predictors. The focus shifted to patients failing to achieve CR following induction therapy. Early events in this subgroup warranted a 12-month time point for analysis. A total of 223 patients were included, with censoring at 12 months. The cohort encompassed patients with progression, relapse, or PR requiring second-line therapy and auto-HSCT within 12 months. Patients under observation without reaching the 12-month mark were excluded.

Patients with extramediastinal involvement had higher risk of poor outcomes compared to those without involvement (OR = 2.26, 95% CI: 1.08–4.73, *P* = 0.025). The presence of bulky tumors (*n* = 212) was associated with a significantly higher risk of poor outcomes compared to the absence of such tumors (*n* = 11) (*P* = 0.018). Soft tissue or breast tissue involvement (*n* = 51) was associated with a trend toward poorer outcomes compared to absence (*n* = 172), approaching statistical significance (OR = 1.92, 95% CI: 1.01–3.68, *P* = 0.038). AI near the *PD-L1* (9p24.1) was significantly associated with a higher risk of poor outcomes (OR = 3.39, 95% CI: 1.18–9.76, *P* = 0.020). LOH at any of the three loci—9p24.1 (*PD-L1*/*PD-L2*), 16p13.13 (*CIITA*), or 6p21.3 (*HLA*)—was significantly associated with poorer outcomes (OR = 4.56, 95% CI: 1.36–15.29, *P* = 0.009). Among all markers analyzed, AI at these loci demonstrated the highest risk for adverse outcomes following induction therapy.

**Figure 6:**
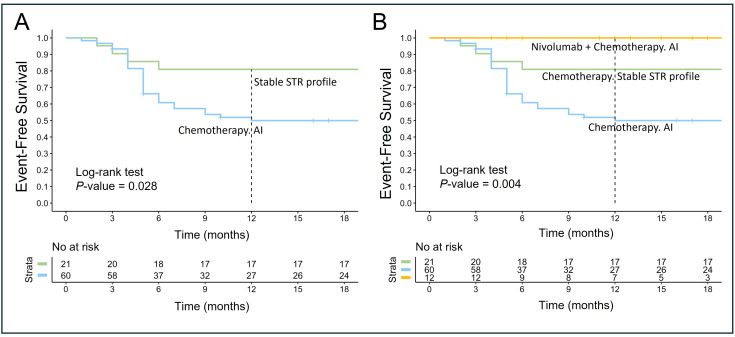
**Event-free survival based on STR profiles 9p24.1 (*PD-L1*/*PD-L2*), 16p13.13 (*CIITA*), 6p21.3 (*HLA*) and first-line therapy**. This figure illustrates EFS in patients with stable STR profile versus those with AI involving 9p24.1 (*PD-L1*/*PD-L2*), 16p13.13 (*CIITA*), and 6p21.3 (*HLA*). (**A**) EFS of patients treated with chemotherapy (R-DA-EPOCH or RmNHL-BFM-90) stratified by STR profile stability. Patients with AI at 9p24.1 (*PD-L1*/*PD-L2*) and/or 16p13.13 (*CIITA*) and/or 6p21.3 (*HLA*) loci had significantly lower EFS compared to those with stable STR profiles (*P* = 0.028); (**B**) EFS of patients stratified by therapy type and STR profiles. Patients treated with nivolumab combined with chemotherapy (nivolumab and R-DA-EPOCH) exhibited no adverse events, achieving 100% EFS despite the presence of AI at the loci analyzed. In contrast, patients receiving chemotherapy alone demonstrated significantly reduced EFS when AI was present (*P* = 0.004). STR: short tandem repeat; *PD-L1*: programmed death ligand-1; *CIITA*: class II, major histocompatibility complex, transactivator gene; *HLA*: human leucocyte antigen; EFS: event-free survival; AI: allelic imbalance; R-DA-EPOCH: rituximab, dose-adjusted etoposide, prednisone, vincristine, cyclophosphamide, doxorubicin; RmNHL-BFM-90: rituximab, modified protocol NHL-BFM-90

Subsequent analysis assessed EFS among patients treated with R-DA-EPOCH or RmNHL-BFM-90 regimens, stratified by STR profiles at 9p24.1, 16p13.13, or 6p21.3 loci. In cases with AI at any locus, EFS decreased to 50% (95% CI: 39–65) compared to 81% (95% CI: 66–100) in patients with stable STR profiles (*P* = 0.028; Figure 6A). In a subgroup of 12 patients treated with nivolumab and R-DA-EPOCH, all exhibited AI in at least one locus, yet no adverse events were observed, yielding an EFS of 100%. This suggests that nivolumab may mitigate the negative impact of AI (*P* = 0.004; Figure 6B).

### Effectiveness of nivolumab in R/R PMBCL

**Figure 7:**
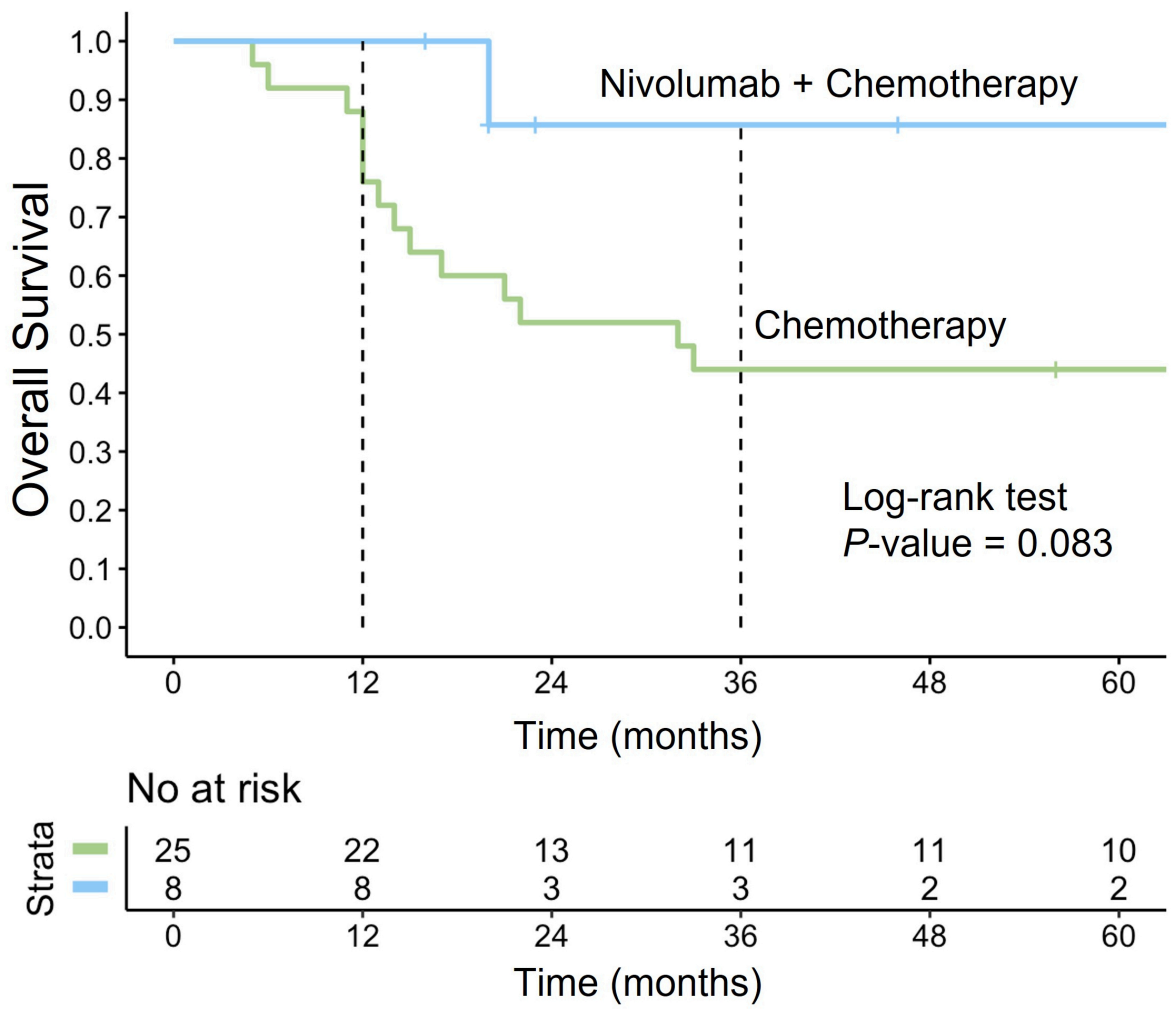
**Overall survival in R/R PMBCL treated with nivolumab plus chemotherapy vs. chemotherapy alone**. The Kaplan-Meier survival curve illustrates the OS in patients with R/R PMBCL treated with nivolumab in combination with chemotherapy (blue curve) compared to those receiving chemotherapy alone (green curve). R/R: relapsed or refractory; PMBCL: primary mediastinal large B-cell lymphoma; OS: overall survival

We analyzed the OS outcomes in a cohort of 33 patients with R/R PMBCL treated with different regimens. Specifically, we compared OS between patients receiving nivolumab in combination with chemotherapy (*n* = 8) and those treated with chemotherapy alone (*n* = 25). The addition of nivolumab demonstrated a clear trend toward improved OS at 36 months, with survival rates of 86% (95% CI: 63–100) in the nivolumab group compared to 44% (95% CI: 28–68) in the chemotherapy-only group (Figure 7). Despite this clinically meaningful difference, statistical significance was not achieved (*P* = 0.083), likely due to the small sample size. The choice of chemotherapy in R/R cases was determined by the patient’s performance status. For most patients, platinum-containing regimens were selected, including R-DHAP (*n* = 17, 52%) and rituximab, ifosfamide, carboplatin, etoposide (R-ICE) (*n* = 6, 18%). Additionally, seven (21%) patients received rituximab, dexamethasone, carmustine, etoposide, cytarabine, melphalan (R-DEXA-BEAM). Two (6%) patients were treated with R-CHALD (rituximab, chlorambucil, etoposide, methotrexate, dexamethasone) and one (3%) with R-GIDOX (rituximab, gemcitabine, ifosfamide, dexamethasone, oxaliplatin).

## Discussion

We observed significant improvement in EFS among patients treated with nivolumab combined with R-DA-EPOCH as a first-line therapy. This suggests the potential of early ICI integration to reduce the need for second-line therapies and auto-HSCT.

A major challenge in the management of PMBCL is the lack of robust predictive markers at disease onset to identify patients who would benefit from early therapy intensification. Risk-adapted strategies are constrained by the absence of universally recognized predictive markers, even with standard R-DA-EPOCH therapy. Earlier studies on low-intensity regimens, such as rituximab, cyclophosphamide, doxorubicin, vincristine, prednisone (R-CHOP), demonstrated that the mutational status of *CD58* gene was associated with inferior PFS (*P* < 0.001) and OS (*P* = 0.02) [10]. In our study, mutations in *CD58* gene were identified in 38% PMBCL patients treated with R-DA-EPOCH or high-dose chemotherapy and did not influence survival outcomes. Our findings associate extramediastinal involvement with an increased risk of early relapse. The ability of nivolumab to cross the blood-brain barrier presents a promising approach for reducing the risk of CNS relapse risk in future studies [43].

Gene expression profiling and next-generation sequencing have shown that overexpression of *PD-L1* and *PD-L2* correlates with poor prognosis in PMBCL (HR 8.2), particularly in protocols such as rituximab, doxorubicin, cyclophosphamide, vindesine, bleomycin, and prednisone (R-ACVBP) and R-CHOP [44]. In our study, AI near the *PD-L1* was associated with inferior EFS. Our research provides novel insights into the analysis of microsatellite aberrations and AI near *PD-L1*/*PD-L2* (9p24.1) and *CIITA* (16p13.13) loci. These findings underline the potential of these markers to refine risk stratification and therapeutic decision-making. However, our data also suggest that nivolumab, when incorporated into first-line therapy, may mitigate the adverse prognostic impact of aberrations at these loci.

Our team has extensive experience using microsatellite markers for oncohematological diagnostics [45], primarily for chimerism monitoring following allogeneic hematopoietic stem cell transplantation. We have frequently observed STR allele loss at relapse or even retrospectively at disease onset [46]. These findings underscore the chromosomal aberrations manifest as AI, reflecting the underlying genomic instability characteristic of PMBCL and other hematologic malignancies [47, 48]. In prior studies, we demonstrated frequent STR profile aberrations in PMBCL compared to DLBCL, indicating a higher prevalence of genomic instability at loci 9p24.1 and 16p13.13 in PMBCL [41, 49]. AI at 6p21.3, while not impactful as an isolated factor in our study, showed a synergistic adverse effect when combined with AI at 16p13.13 and 9p24.1, leading to poorer EFS. These findings emphasize the importance of integrated molecular diagnostics to uncover multi-locus aberrations that may influence treatment response and outcomes.

In summary, our findings support the early integration of ICIs into the treatment strategy for PMBCL, particularly in patients with a high risk of recurrence. This approach has the potential to counteract unfavorable prognostic factors and enhance long-term outcomes, although further prospective studies are necessary to confirm these observations.

### Limitations

The present study has several limitations that must be acknowledged. The small sample size constrained the statistical power of some analyses, particularly in subgroup comparisons, calling for cautious interpretation of the results. This was especially relevant for the immunotherapy cohort, where the limited number of patients and observed events restricted the robustness and generalizability of the findings. Future research should focus on long-term follow-up to evaluate the durability of responses and survival outcomes in patients receiving ICIs. Additionally, exploring combination strategies, such as integrating immunotherapy with novel targeted agents, holds promise for improving outcomes in high-risk PMBCL subsets. To validate the current findings and further refine treatment approaches, larger, prospective studies are needed to provide more conclusive evidence and support personalized, risk-adapted strategies in PMBCL management.

### Conclusions

The findings emphasize the importance of integrating ICIs, such as nivolumab, into first-line treatment for PMBCL patients, particularly those with high-risk clinical features. Furthermore, the strong prognostic role of AI at key loci (9p24.1, 16p13.13, 6p21.3) underscores the need for routine molecular profiling to guide risk-adapted treatment strategies. To validate these findings, larger, prospective studies with extended follow-up are required. Additionally, further exploration of ICIs in both first-line and R/R settings, combined with molecular characterization, will refine personalized treatment approaches and improve outcomes for patients with PMBCL.

## Abbreviations

AI: allelic imbalance
auto-HSCT: autologous hematopoietic stem cell transplantation
*B2M*: beta-2-microglobulin
*BCL2*: B-cell lymphoma 2
CI: confidence intervals
*CIITA*: class II, major histocompatibility complex, transactivator gene
CMA: chromosome microarray analysis
cnLOH: copy-neutral loss of heterozygosity
CR: complete remission
CTLA-4: cytotoxic T-lymphocyte-associated protein 4
DLBCL: diffuse large B-cell lymphomas
EFS: event-free survival
ER: epitope retrieval
HLA: human leucocyte antigen
ICIs: immune checkpoint inhibitors
IHC: immunohistochemical
*MHC*: major histocompatibility complex
MUM1: multiple myeloma oncogene 1
*MYC*: myelocytomatosis oncogene
OR: odds ratios
OS: overall survival
PCR: polymerase chain reaction
PD-1: programmed death-1
PD-L1: programmed death ligand-1
PFS: progression-free survival
PMBCL: primary mediastinal large B-cell lymphoma
PR: partial remission
R/R: relapsed or refractory
R-DA-EPOCH: rituximab, dose-adjusted etoposide, prednisone, vincristine, cyclophosphamide, doxorubicin
RmNHL-BFM-90: rituximab, modified protocol NHL-BFM-90
STR: short tandem repeat
*TP53*: tumor protein p53
*XPO1*: exportin 1

## Declarations

### Availability of data and materials

The raw data supporting the conclusions of this manuscript will be made available by the authors, without undue reservation, to any qualified researcher.

### Copyright

© The Author(s) 2025.
